# Supplementary figures and images for: V-ATPase is a universal regulator of LC3-associated phagocytosis and non-canonical autophagy
Source: J Cell Biol. 2022 May 5;221(6):e202105112. doi: 10.1083/jcb.202105112 (PMC9082624; doi:10.1083/jcb.202105112)

Source Data Figure 5C

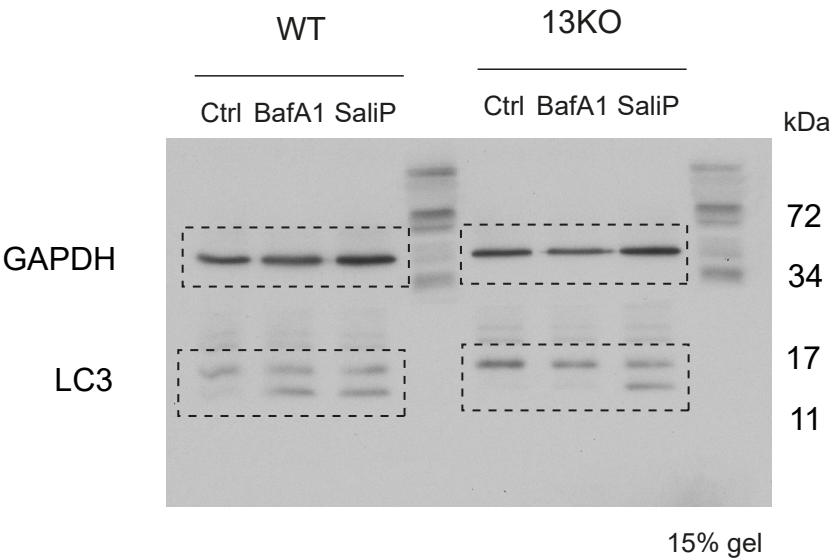

Supplement: SourceData F5 — contains original blots for Fig. 5. [file JCB_202105112_SourceDataF5.pdf]

Source Data Figure 6

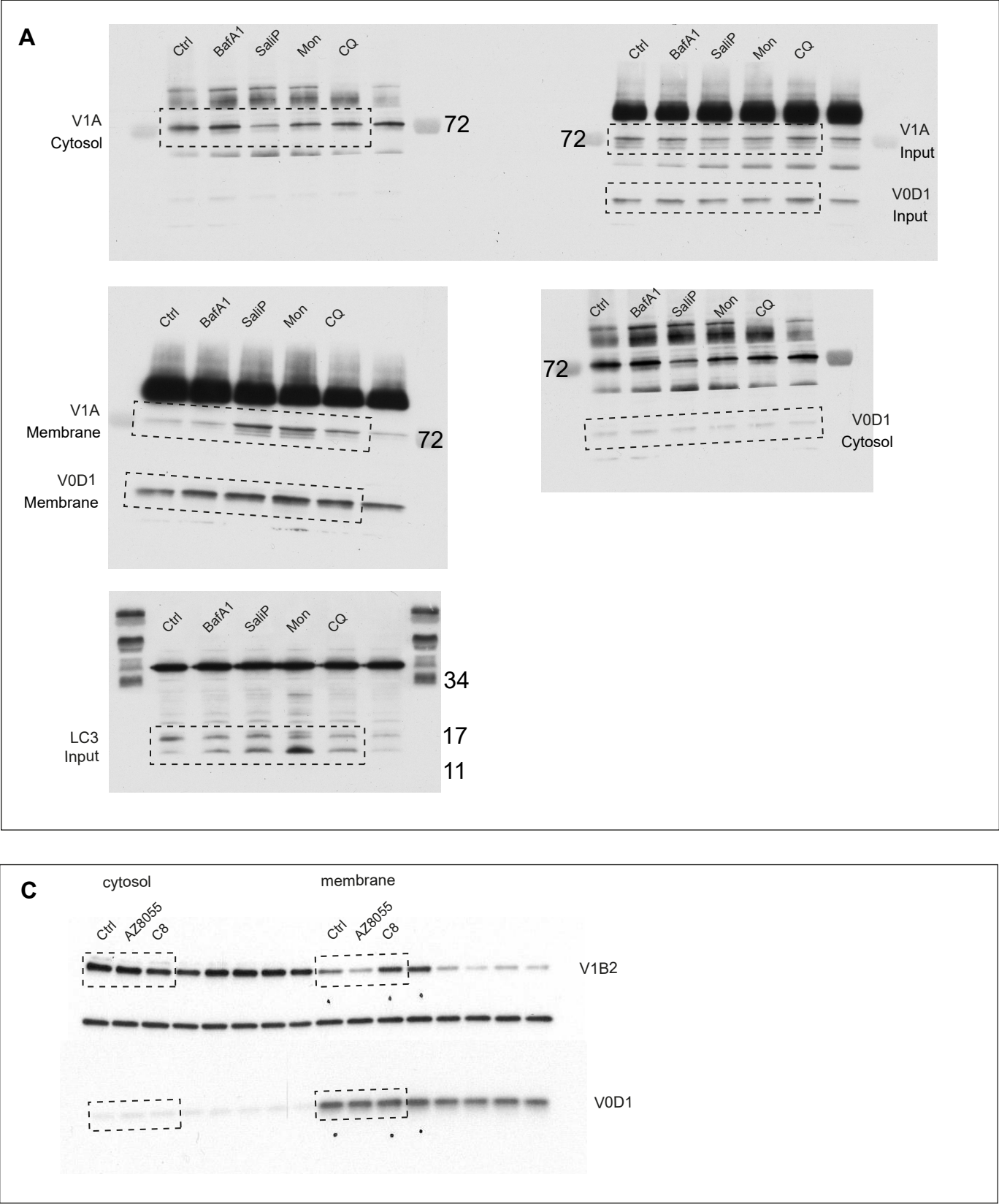

Supplement: SourceData F6 — contains original blots for Fig. 6. [file JCB_202105112_SourceDataF6.pdf]

Source Data Figure 7

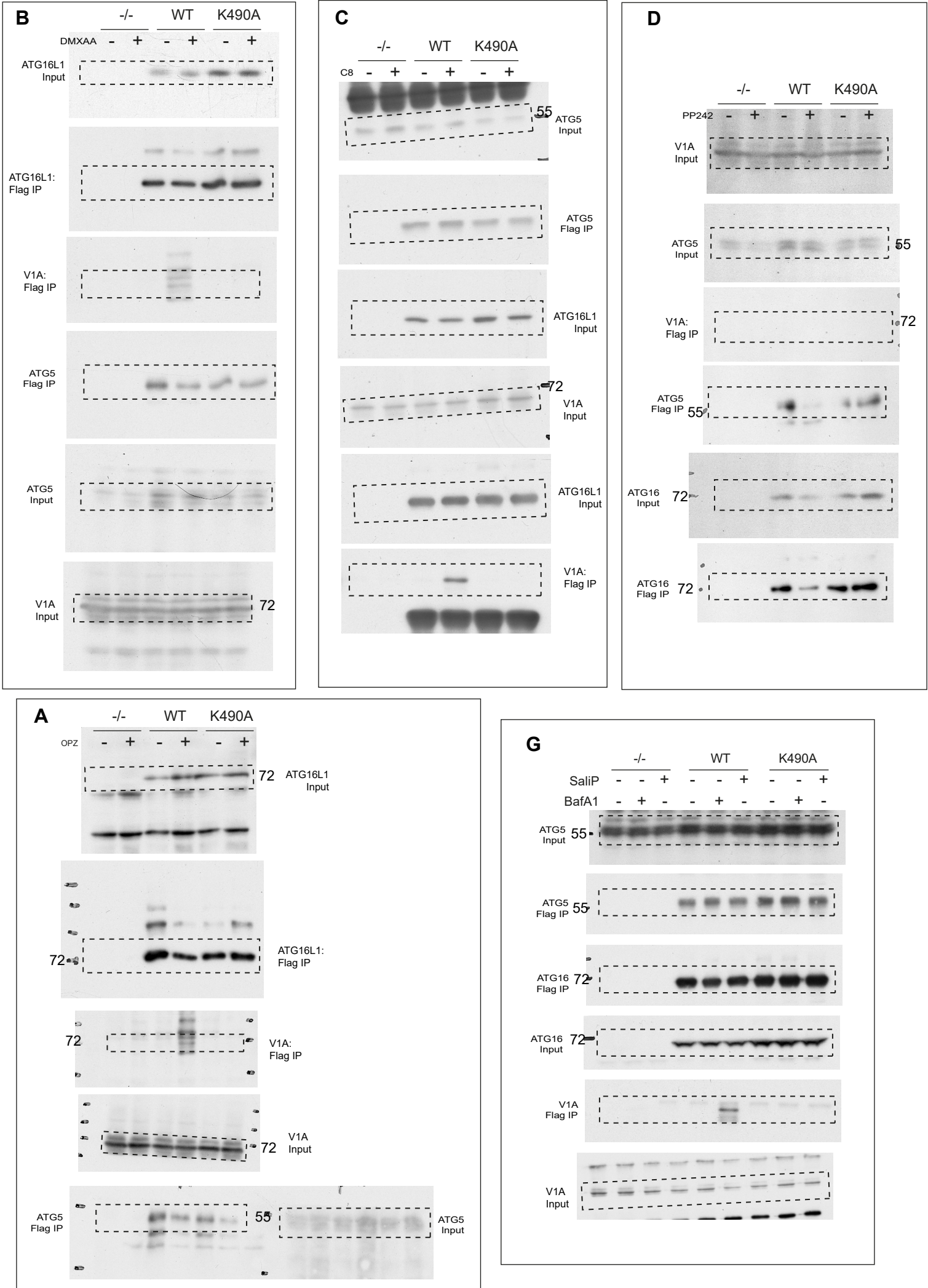

Supplement: SourceData F7 — contains original blots for Fig. 7. [file JCB_202105112_SourceDataF7.pdf]

Source Data Figure 8D

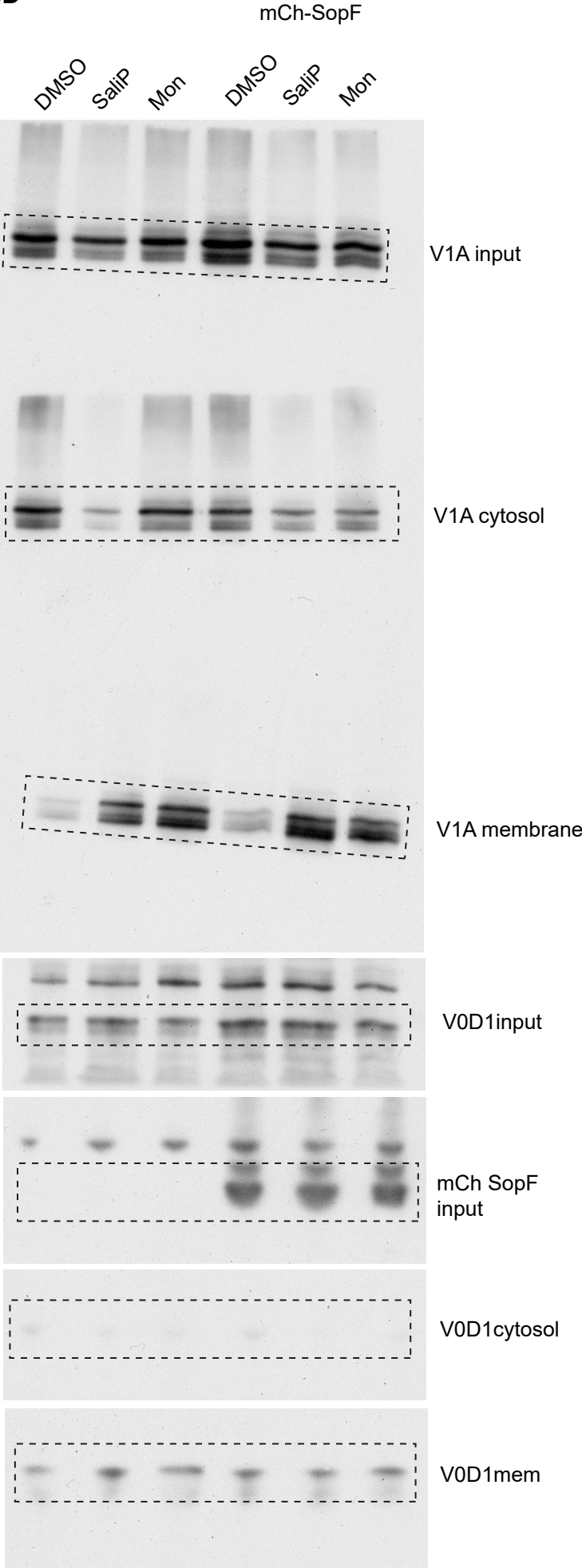

Supplement: SourceData F8 — contains original blots for Fig. 8. [file JCB_202105112_SourceDataF8.pdf]

Source Data Figure S4

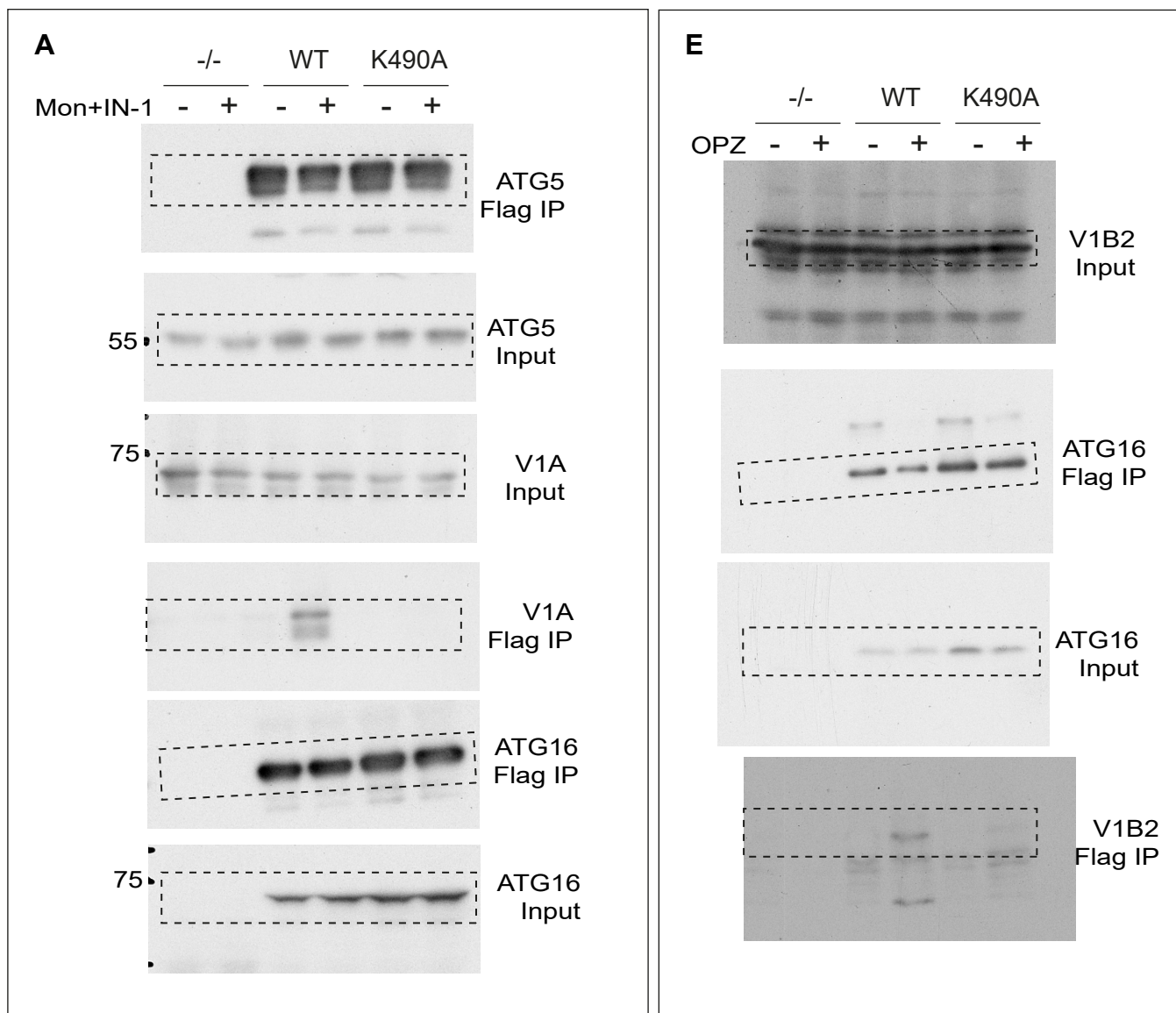

Supplement: SourceData FS4 — contains original blots for Fig. S4. [file JCB_202105112_SourceDataFS4.pdf]
